# Quantification and significance of extraprostatic findings on prostate MRI: a retrospective analysis and three-tier classification

**DOI:** 10.1186/s13244-023-01549-9

**Published:** 2023-12-10

**Authors:** Monika Wagnerova, Iva Macova, Petr Hanus, Martin Jurka, Otakar Capoun, Lukas Lambert, Andrea Burgetova

**Affiliations:** 1 Department of Radiology, First Faculty of Medicine, Charles University and General University Hospital in Prague, Prague 2, 128 08 Czech Republic; 2 Department of Urology, First Faculty of Medicine, Charles University and General University Hospital in Prague, Prague 2, 128 08 Czech Republic

**Keywords:** Magnetic resonance imaging, Prostate, Cancer, PI-RADS, Extraprostatic findings

## Abstract

**Objectives:**

To quantify extraprostatic findings (EPFs) on prostate MRI, estimate the proportion of reported and unreported EPFs, assess their clinical importance, and propose standardized reporting of EPFs.

**Materials and methods:**

Prostate 3-T MRI studies, reports, and clinical data from 623 patients (age 67.9 ± 8.2 years) were retrospectively analyzed and re-evaluated for the presence of EPFs and their clinical significance: E1—no finding or findings that have no clinical significance; E2—potentially significant findings; and E3—significant findings.

**Results:**

Secondary reading identified 1236 EPFs in 593 patients (1.98 ± 1.13 EPFs per patient, no EPFs in 30 patients), from which 468 (37.8%) were mentioned in the original report. The most common findings included diverticulosis (44% of patients), hydrocele (34%), inguinal fat hernia (16%), and bladder wall trabecular hypertrophy (15%). There were 80 (6.5%) E2 EPFs and 30 (2.4%) E3 EPFs. From E3 EPFs, 10 (33%) were not originally reported. A workup was suggested in 35 (52%) of the 67 originally reported E2 and E3 findings with follow-up and performed in 20 (30%). Fourteen (21%) EPFs in 11 patients influenced their management. Four experienced radiologists originally reported 1.8 to 2.5 findings per patient (*p* < 0.0001).

**Conclusions:**

EPFs on prostate MRI are frequent, but only 2.4% are clinically significant (E3), and 33% of these are not reported. Only 30% of E2 and E3 findings are further explored, and 21% influence patient management. We suggest that an “E” category should be attached to the PI-RADS system to identify the presence of EPFs that require further workup.

**Critical relevance statement:**

Extraprostatic findings on prostate MRI are frequent, but only 2.4% are clinically significant (E3), and 33% of these are not reported. We advocate standardized reporting of extraprostatic findings indicating their clinical significance.

**Key points:**

• Extraprostatic findings on prostate MRI are frequent with an average of two findings per patient.

• 2.4% of extraprostatic findings are significant, and 33% of these are not reported.

• There is a significant variability among experienced radiologists in reporting extraprostatic findings.

**Graphical Abstract:**

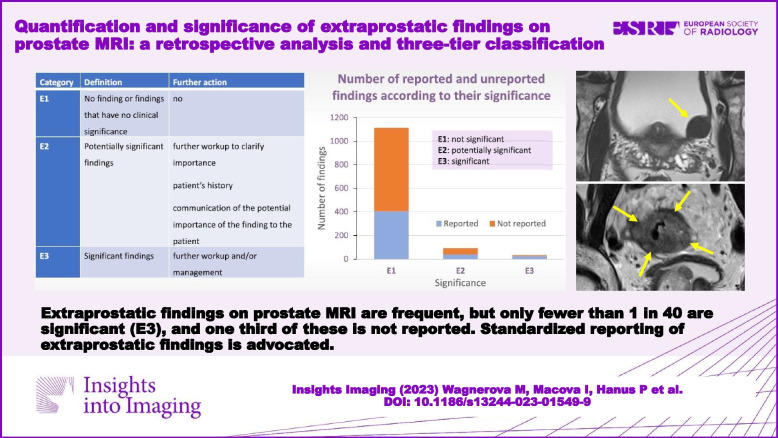

**Supplementary Information:**

The online version contains supplementary material available at 10.1186/s13244-023-01549-9.

## Introduction

Magnetic resonance imaging (MRI) of the prostate improves the detection of clinically significant prostate cancer and reduces overdiagnosis and the number of biopsy procedures compared to systematic biopsy alone [1, 2]. Extraprostatic findings (EPFs) are abnormalities detected outside of the prostate gland. Their impact on patient’s life is mostly negligible. Only a small proportion of incidental findings on prostate MRI have clinical relevance or require further workup [3].

Prostate Imaging Reporting and Data System (PI-RADS®) provides a standardized method for interpreting MRI of the prostate [4]. PI-RADS uses a scoring system from 1 to 5, with higher scores indicating a higher likelihood of clinically significant prostate cancer. PI-RADS does not standardize the reporting of EPFs. Unlike in the CT-Colonography Reporting and Data System (C-RADS), where extracolonic findings have a separate “E” category, EPFs are reported separately and without explicit clarification of their potential clinical significance [5]. The C-RADS E-score was designed to standardize the reporting of extracolonic findings to avoid unnecessary processing of clinically unimportant findings, efficiently communicating the need for workup of potentially important findings for the benefit of patient’s outcome [6]. Considering the proposed population screening for prostate cancer including prostate MRI in the Czech Republic, it is necessary to describe the occurrence of EPFs quantitatively and assess their clinical relevance.

Previous studies have reported the types and frequency of EPFs on prostate MRI with different imaging protocols, from primary reports or secondary readings, and in different age populations [3, 7, 8]. The frequency and spectrum of EPFs and the interpretation of their clinical relevance have been subject to variability. The heterogeneity between primary and secondary reading focused on EPFs has not been quantified.

The aims of this study were to (1) quantify EPFs on prostate MRI, (2) estimate the mismatch between reported and unreported EPFs, (3) assess the clinical importance of EPFs, and (4) propose standardized reporting of EPFs.

## Methods

This retrospective study was carried out in agreement with the Declaration of Helsinki (ver. 2013). The Ethics Committee of the General University Hospital in Prague stated that the study required neither its approval nor informed consent.

### Patient population

**Fig. 1:**
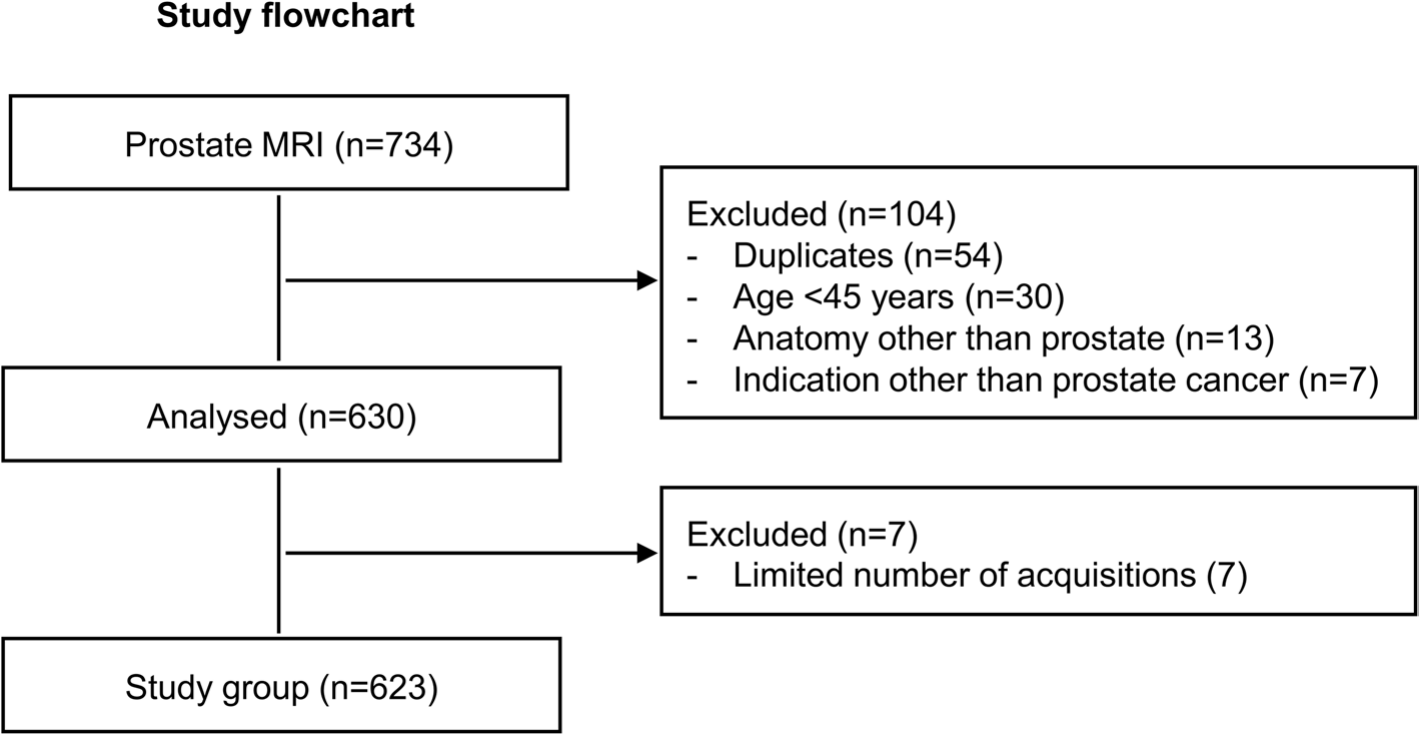
Study flowchart

We included a patient population of consecutive subjects ≥ 45 years of age who underwent biparametric or multiparametric prostate MRI for suspected or known prostate cancer including diffusion-weighted imaging (DWI), T1-weighted imaging in the axial plane, and T2-weighted imaging in at least two planes performed between January 2021 and December 2022 in a tertiary academic hospital [9]. The flow diagram is shown in Fig. 1.

### MR acquisition

The examinations were performed on two 3-T scanners, Ingenia Elition (Philips) and Magnetom Skyra (Siemens), using phased array coils. During the study interval, the imaging protocol on the first scanner was updated. A protocol that included large field of view (FOV) sagittal or transverse acquisitions (P1) on the first scanner was superseded by a protocol with limited craniocaudal coverage (P2). The imaging protocol on the second scanner remained unchanged throughout the study period (P3). The protocols are compared in Table 1.

**Table 1:** Protocols used during the study period

Protocol	Scanner	Patients, *n*	Age, years ± SD	Feet-head coverage	Large FOV	Limited FOV	DCE, *n* (%)	Findings per patient
P1	Ingenia Elition	77	68.0 ± 7.8	Kidney-perineum	T2SSh sag, T1 DIXON tra	T2 tra, T2-3D, DWI (± DCE)	3 (3.9%)	2.6 ± 1.2
P2	Ingenia Elition	128	68.7 ± 7.6	Pelvic rim-perineum	T1 DIXON tra	T2 tra, T2 sag, T2-3D (± DCE)	7 (5.5%)	2.3 ± 1.1
P3	Magnetom Skyra	418	67.9 ± 8.4	Kidney-perineum	True-FISP sag	T2 tra, T2 sag, T2 cor (± DCE)	412 (98.5%)	1.8 ± 1.1
*p* value			0.60				< 0.0001^a^	< 0.0001^b^

*FOV*, field of view; *T2SSh*, T2 weighted single-shot sequence; *DIXON*, T1 weighted gradient echo sequence; *True-FISP*, T2 weighted sequence with steady-state precession; *DWI*, diffusion-weighted imaging; *DCE*, dynamic contrast enhancement; *tra*, transverse plane; *cor*, coronal plane; *sag*, sagittal plane

^a^Significant between P1 vs. P3 and P2 vs. P3

^b^Post hoc tests significant for P1 vs. P3 and P2 vs. P3

### MR evaluation and data processing

MRI reports and clinical data were obtained from the hospital information system. MRI reports were searched for PI-RADS scores, prostate volume, and reported EPFs. Clinical records were analyzed to determine whether reported EPFs had been acted upon, and what the final importance of these findings was.

MRI studies were retrieved from the local picture archiving and communication system (PACS) and reviewed on a clinical workstation. Secondary reading was split among six radiologists with experience in MRI (B.A., 16 years of experience; H.P., 8 years; J.M., 5 years; L.L., 9 years; M.I., 3 years; W.M., 10 years).

The radiologists reported EPFs and assigned them a clinical significance grade using a three-tier system (Table 2), which was based on previous studies and the C-RADS classification [5–7]. A list of examples was added.

**Table 2:** Proposed classification of extraprostatic findings

Category	Definition	Examples	Further action
E1	No finding or findings that have no clinical significance	Small to moderate hydrocele Mild to moderate diverticulosis Degenerative spine disease, coxarthrosis gr. I to II Enthesopathy Preperitoneal lipoma Mild to moderate trabecular hypertrophy of the bladder wall	No
E2	Potentially significant findings	Large hydrocele Severe diverticulosis Degenerative spine disease with absolute spinal stenosis, nerve compression Severe coxarthrosis (gr. III to IV), enthesopathy, bursitis Inguinal hernia containing bowel loops Marked trabecular hypertrophy of the bladder wall, bladder diverticula	Further workup to clarify importance Patient’s history Communication of the potential importance of the finding to the patient
E3	Significant findings	Aneurysm or occlusion of the aorta, iliac artery Diverticulitis Hydronephrosis Tumors Osteolytic or osteoblastic bone lesions Stones in the bladder or ureter	Requires further workup and/or management

To assess the interobserver agreement for the proposed classification, 3 raters (W.M., M.I., L.L.) independently evaluated the E category in a subset of 53 randomly selected patients after a 3-month period to avoid recall bias.

### Statistical analysis

Statistical analysis was performed in R (R Foundation, Austria, Vienna) and Prism (GraphPad Software, La Jolla, CA). Dichotomous data were compared using the *χ*^2^ test, Fisher test, continuous and ordinal data using the Mann–Whitney test, Kruskall-Wallis test with Dunn’s post hoc tests, and one-way ANOVA with Bonferroni’s post hoc tests and expressed as median (interquartile range, IQR) or average ± standard deviation according to their distribution (D’Agostino and Pearson omnibus normality test). Correlations were calculated using Spearman rank correlation (rho, *ρ*). For age adjustment, a *ppcor* function in R was used. Interobserver agreement for the proposed classification was calculated using the *KappaM* function in R as Fleiss’ kappa (extension of Cohen’s kappa for > 2 raters). A *p*-value < 0.05 was considered significant.

## Results

The final study sample comprised 623 male patients (age 67.9 ± 8.2 years) who underwent prostate MRI for suspected or known (active surveillance, radiotherapy planning) prostate cancer between January 2021 and October 2022. A study flowchart is shown in Fig. 1.

There were 13 patients after radical prostatectomy, 35 patients after transurethral resection of the prostate (TURP), and 1 after transvesical prostatectomy. Prostate volume was 52.0 (IQR 36.5–73.0 ml). PI-RADS ≤ 2 was reported in 309 (49.6%) patients and PI-RADS ≥ 3 in 290 (46.5%) patients; in 24 (3.9%) patients, PI-RADS was not reported.

**Fig. 2:**
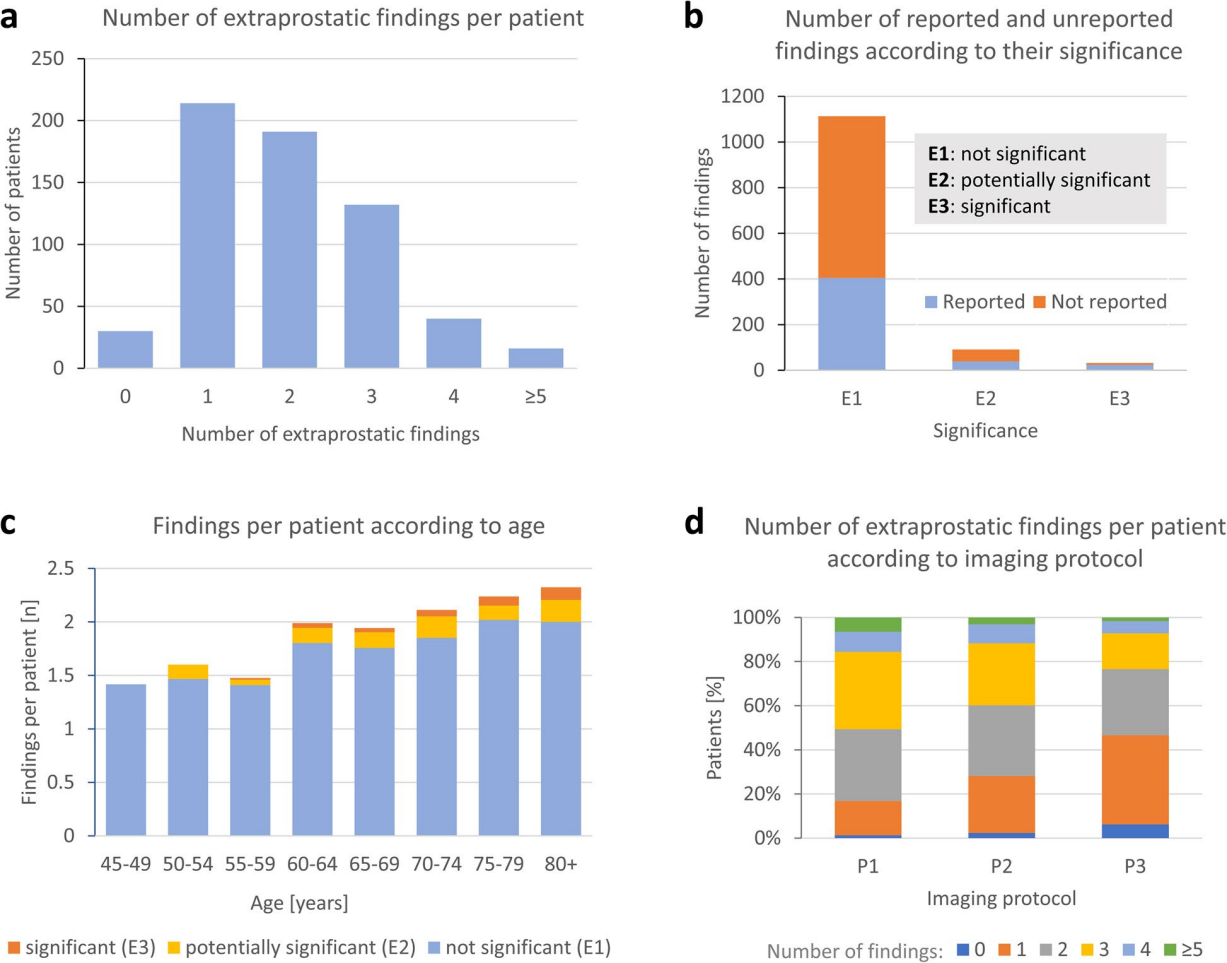
Number of extraprostatic findings per patient (**a**). Number of reported and unreported findings according to their significance (**b**). Number of findings per patient according to age stratified by their significance (**c**). Number of extraprostatic findings according to the imaging protocol (P1, P2, and P3; **d**). P2 denotes protocol with limited anatomical coverage

Protocol P1 had the highest number of findings per patient (2.6 ± 1.2), and protocol P3 had the lowest (1.8 ± 1.1, *p* < 0.00011, Table 1, Fig. 2d). No EPF was reported in 30 (4.8%) patients.

Patients with PI-RADS 5 lesions were older (72.0 ± 6.2 years, *p* < 0.0001) and had a higher number of findings per patient (2.3 ± 1.3, *p* = 0.0053, Additional file 1: Table S1, Fig. 2c).

There was a significant correlation between the highest PI-RADS category and the number of findings (*ρ* = 0.011, *p* = 0.010) or age (*ρ* = 0.18, *p* < 0.0001). Age corrected correlation between the highest PI-RADS category, and the number of findings was marginally significant (*ρ* = 0.083, *p* = 0.042).

Secondary reading identified 1236 extraprostatic findings in 593 patients (Fig. 2a), from which 468 (37.8%) were mentioned in the original report (Fig. 2b). No extraprostatic findings were found in 30 (4.8%) patients. The average number of EPFs per patient was 1.98 ± 1.13. The most common findings were diverticulosis in 44% of patients followed by hydrocele in 34%, inguinal fat hernia in 16%, and bladder trabecular hypertrophy in 15% of patients (Table 3, Additional file 1: Table S2).

**Table 3:** Frequent and common extraprostatic findings (> 1% of patients) sorted by their frequency

Anatomy	Condition	Frequency, % of 623 pts	Non-significant, no	Potentially significant, no	Significant, no	Reported, no	Not reported, no.^a^	Percent, %
**FREQUENT**	** ≥ 5%**							
**Bowel**	Colon diverticulosis	44.3	266	10		130	146	47.1
**G/U**	Hydrocele	34.0	207	5		44	168	20.8
**Abd. wall**	Inguinal hernia (fat)	15.6	95	2		25	72	25.8
**G/U**	Trabecular hypertrophy	14.6	88	2	1	37	54 (0)	40.7
**MSK**	Coxartrosis (gr. III or IV)	9.5	46	11	2	23	36 (2)	39.0
**MSK**	Alloplasty, osteosynthesis	5.8	36			24	12	66.7
**MSK**	Foraminostenosis with contact or compression	5.3	32	1		3	30	9.1
**COMMON**	** > 1%**							
G/U	Cyst simple	4.5	28			12	16	42.9
MSK	Tendinosis or enthesopathy	4.2	20	6		1	25	3.8
G/U	Bladder diverticulum	3.9	23	1		13	11	54.2
MSK	Tarlov cyst	3.4	21			4	17	19.0
MKS	Bursitis	3.2	19	1		9	11	45.0
G/U	Blood in seminal vesicle	3.2	19	1		13	7	65.0
MSK	Hip periarticular changes	3.0	19			3	16	15.8
MSK	BM focal changes	2.6	7	4	5	11	5 (1)	68.8
G/U	Cyst—testis, epidydimis	2.6	15	1		8	8	50.0
Lymphatic	Enlarged lymph nodes	2.6	5	3	8	12	4 (2)	75.0
Abd. wall	Umbilical hernia—fat	2.1	12	1		3	10	23.1
MSK	Hip—focal changes^b^	1.9	11		1	3	9 (1)	25.0
Peritoneum	Ascites	1.9	12			6	6	50.0
MSK	Degenerative changes of symphysis	1.6	9	1		3	7	30.0
Vascular	Atherosclerosis (> 50% stenosis)	1.6	6	4		1	9	10.0
MSK	Mass (lipoma, cyst, tumor)	1.3	7		1	3	5 (1)	37.5
G/U	Stone	1.3	3	5		6	2	75.0

*G/U*, genitourinary; *BM*, bone marrow; *MSK*, musculoskeletal

^a^Number in parentheses denotes unreported significant findings

^b^Non-degenerative

**Fig. 3:**
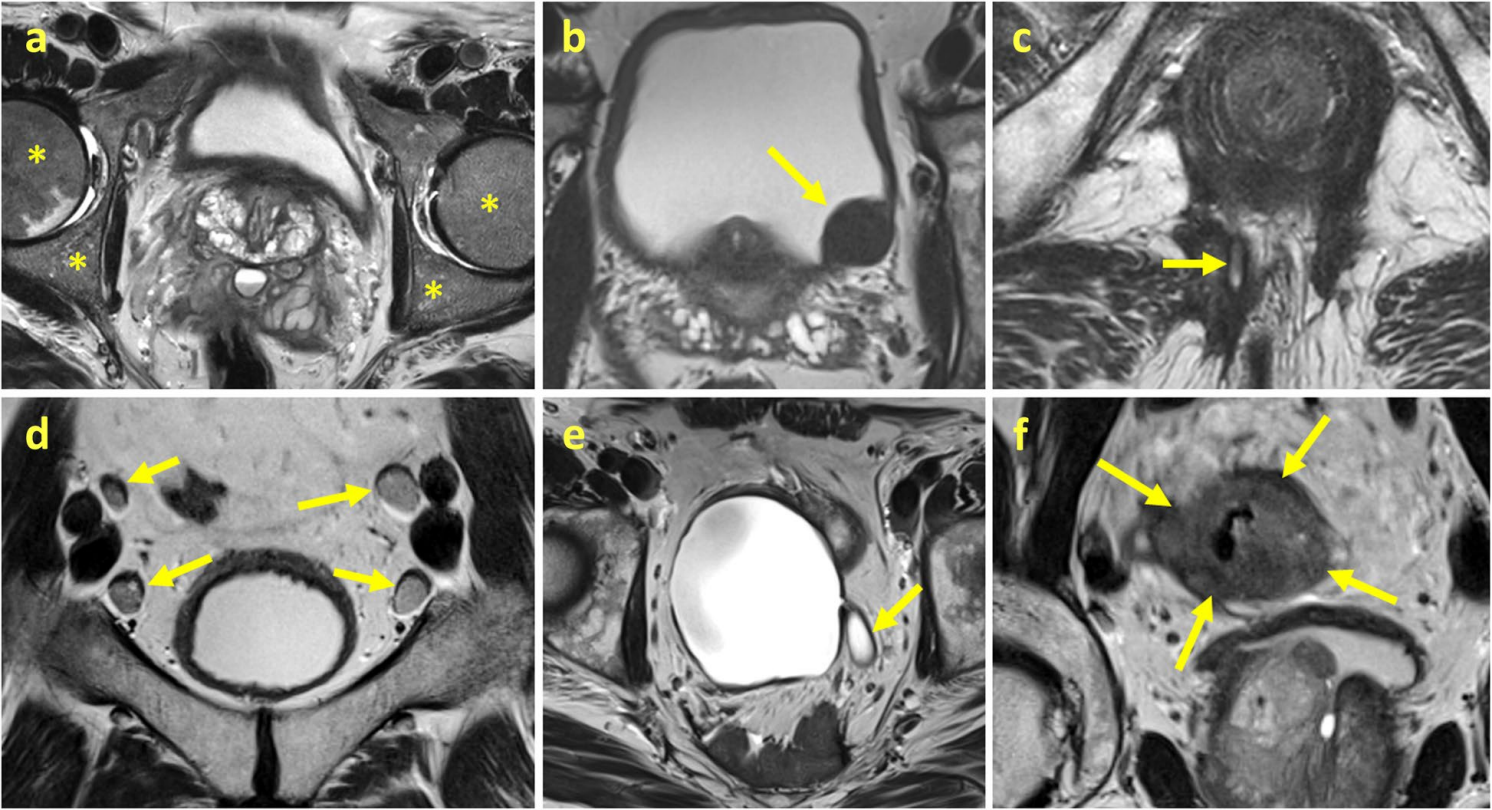
Examples of significant findings that were not included in the original report (T2-weighted fast spin echo). Diffuse bone marrow changes (**a**). Bladder wall tumor (**b**). Perianal fistula (**c**). Lymphadenopathy (**d**). Hydroureter (**e**). Sigmoid cancer (**f**)

There were 80 (6.5%) potentially clinically significant EPFs (*E2*) identified on the secondary reading. Of the 30 (2.4%) clinically significant EPFs (*E3*), 10 (33%) were not mentioned in the original report (Fig. 2b). The most underreported significant findings included severe coxarthrosis (2 patients), indeterminate lymphadenopathy (2 patients), focal bone marrow changes, focal changes in the hip, soft tissue tumor, diffuse bone marrow changes, hydronephrosis, and cavernous body thrombosis (1 patient each, Fig. 3).

The proportion of findings originally reported by four readers (R1 to R4, Additional file 1: Table S3), who read > 50 MRI included in the analysis ranged from 24 to 51% with 1.8 to 2.5 findings per patient (*p* < 0.0001, post hoc significant R1–R3 and R2–R3). When analyzing the most common findings, the highest difference in reporting was noted for hydrocele (8 to 58%) and coxarthrosis (8 to 100%, Additional file 1: Table S3). The interobserver agreement for the proposed E category was 0.86 (95%CI 0.72 to 0.99).

Clinical follow-up was available for 67 of 71 (94.4%) E2 or E3 findings that were reported on primary reading in 58 patients. These patients had the last clinical visit 248 (IQR 145–614) days after MRI. A workup was suggested in 35 of the 67 (52%) findings and performed in 20 (30%) (Additional file 1: Table S4). There were 14 (21%) findings in 11 patients that changed their management (surgery, pharmacotherapy, anticancer treatment).

## Discussion

In this study, we showed on a sample of 623 patients/MRI examinations that there were on average 1.98 extraprostatic findings (EPFs) per patient, and from these, only one-third were included in the original report. The most common findings were clinically unimportant. Only 2.4% of all findings were considered significant, but 33% of them were not reported. In significant and potentially significant EPFs, workup was suggested in 52%, performed in 30%, and only in 21% influenced patients’ management. There was a significant difference in the findings originally reported by radiologists from 24 to 51% of patients.

Identification of incidental EPFs contributes to the benefits of prostate MRI but also poses the risk of overdiagnosis, which is accompanied by secondary examinations, anxiety, additional cost, and morbidity associated with invasive procedures [10, 11]. We showed that EPFs are common, but only a small fraction of them is clinically significant.

Sherrer et al. [7] found that 349 truly incidental findings were reported in 233 (40%) reports of 580 patients, who underwent multiparametric prostate MRI examinations with 6.6% being considered clinically significant. The number of EPFs in our slightly older population was four times higher (especially diverticulosis, bladder wall trabecular hyperplasia, inguinal fat hernia, and hydrocele). The difference is explained by the fact that radiologists report between 24 and 51% of EPFs as shown in our study. In Sherrer’s study, the protocol included abdominopelvic contrast-enhanced T1, which could explain a higher number of significant findings (renal mass, liver lesion, aneurysm). On the other hand, the number of patients with benign scrotal pathology (hydrocele) was lower. Our study confirmed their observation that diverticulosis, fat-containing inguinal hernia, and trabecular hypertrophy are the most common EPFs.

Cutaia et al. performed a secondary reading of multiparametric prostate MRI and reported fewer EPFs (461 in 647 patients) that were present in 53% of patients compared to 95% of patients in our study [3]. The number of significant findings was comparable. The most common findings related to the genitourinary system were bladder diverticula followed by trabecular bladder wall hypertrophy. In our study, the frequency of trabecular bladder wall hypertrophy clearly dominated the frequency of bladder diverticula. The proportion of patients with colonic diverticulosis was 19% compared to 44% in our study, which could reflect the difference in the prevalence between the Italian and Czech populations. Their scope of EPFs was similar including more musculoskeletal findings, which is the result of secondary reading focused on EPFs.

In another study, Ediz and Gunduz [8] detected on secondary reading EPFs in 44 of 185 patients (48%) who underwent multiparametric prostate MRI. They identified inguinal hernias (28% of all patients), bladder wall abnormalities (16%), hydrocele (14%), and sigmoid diverticula (7%) as the most common EPFs. Again, the striking difference in the frequency of diverticulosis can be explained by lower disease prevalence in the Turkish compared to Czech population [12]. The proportion of significant findings was comparable. But apart from Tarlov cysts, Ediz and Gunduz did not report any other musculoskeletal findings.

In comparison with the previously published literature on the prevalence of EPFs in patients undergoing prostate MRI, we additionally quantified the difference between the frequency of EPFs that were mentioned in the original report (a strategy used by Sherrer et al. [7]) and that were found on secondary targeted reading [3, 8]. Heterogeneity in reporting EPFs in primary reports has been recognized. Also, we identified more types of EPFs, which may serve as a hint to radiologists about what else can be found beyond the prostate (numerous musculoskeletal findings, significant arterial stenosis) and explain the patient’s complaints unrelated to the prostate. However, even our list is not complete. A systematic pictorial checklist has been published by Ponsiglione et al., who also categorize EPFs as significant and not significant [13]. It should be noted that findings commonly without significance may become significant due to their size or severity.

The number of EPFs increases with age. Also, a higher PI-PRADS category is more common in older age, and these patients have more EPFs, as has been shown previously [3, 6, 7].

The present study showed heterogeneity in reporting EPFs among radiologists who regularly report prostate MRI as it has been reported in PI-RADS itself [14]. Although protocol P2 with limited coverage had a lower yield of EPFs than P1, the least EPFs were reported from protocol P3 with full coverage because one radiologist had a higher threshold for including EPFs in the report and rarely mentioned insignificant EPFs. There are no criteria for grading the severity of hydrocele, diverticulosis, trabecular hypertrophy of the bladder wall, and many other findings on imaging. It is noteworthy that in the case of potentially significant and significant EPFs (E2 and E3), workup was suggested in 52%, performed in 30%, and in only 21% it influenced patients’ management.

On the secondary reading, protocol P3 had the lowest number of extraprostatic findings. The sagittal True-FISP sequence in P3 features band-like artifacts in the periphery, limiting cranio-caudal coverage. Also, most patients examined with P3 were referred from our tertiary center, whereas the majority of patients examined with P1 and P2 came from private clinics representing slightly different populations.

Radiologists should search for potentially significant EPFs related not only to the genitourinary system such as marked bladder wall trabecular hyperplasia, hydrocele, and tumors of the urinary bladder and seminal vesicles. Potentially significant findings beyond the genitourinary system include focal and diffuse bone marrow changes, soft tissue tumors, colon cancer, inguinal hernia, stenosis of the iliac vessels, grade III to IV coxarthrosis, enthesopathy, bursitis, and degenerative spine disease with spinal stenosis or nerve root compression. For example, marked trabecular hypertrophy of the urinary bladder wall and the presence of diverticula correlate with urinary retention and the need for intervention [15]. There are also very rare findings that have been described previously but not detected in our cohort of patients, including periprostatic leiomyomas, lipomas, fibrous tumors, chordomas, gastrointestinal stromal tumors, and their malignant variants [16, 17]. Clinicians should be advised that prostate MRI also depicts other structures and should be encouraged to request a second reading with a specific question which is occasionally done (mostly for musculoskeletal system and soft tissue).

In CT colonography, the C-RADS classification communicates extracolonic findings and their importance by the E category [5]. We believe that this strategy should be adopted by the PI-RADS classification system as well. A category reporting the absence of EPSs is not necessary (< 5% of patients). We suggest that a three-tier system be adopted. A PI-RADS E1 category should encompass all findings that are frequent and bear no clinical significance such as small hydrocele, mild to moderate diverticulosis, degenerative spine disease, preperitoneal lipoma (fat-containing inguinal hernia), and mild to moderate trabecular hypertrophy of the bladder wall. A PI-RADS E2 category raises the possibility of clinical importance, and the E3 category is imperative for further workup and management (Table 2).

In order to simplify the coding, Yee et al. and Sherrer et al. advocated a two-tiered system that would categorize the findings according to their significance to high clinical significance and low to moderate clinical significance [6, 7]. Cutaia et al. preferred a three-tiered system with non-significant, potentially significant, and significant findings which we also advocate. This system has shown a perfect agreement.

A finding with no clinical importance requires no attention. Further management of significant and potentially significant findings must be considered. First, it is necessary to review the clinical history of the patient, whether the finding is known and has already been negotiated. Second, if the finding is not fully characterized, further tests may be required. Third, the clinical relevance of the finding has to be reviewed through the patient’s complaints and his general condition. Fourth, the significance or non-significance of the finding has to be communicated to the patient. Fifth, in complex cases, a multidisciplinary team or a specialist may need to be consulted. Although there are white papers on managing common incidental findings available [18–20], further work-up must be contemplated with respect to the expected benefit and harm for a particular patient [21]. Specific recommendations can be made for the most common findings, but their description is beyond the scope of this study and spans multiple specialties.

An agreement exists that a level of standardization for a more accurate comparison between studies is needed [6, 7]. We believe that the inclusion of the category of “extraprostatic findings” in the PI-RADS scale, referred to as E in the reports of prostate MRI, and classifying the findings into E1, E2, and E3 based on their clinical relevance and prognostic significance would facilitate decision-making for the requesting physician, as well as the use of a universal language for radiologists and non-radiologist physicians.

Based on this study and our experience, we suggest that a category for the classification of EPFs on prostate MRI should be attached to the PI-RADS classification to red-flag findings that require further workup or management like in the C-RADS E category.

## Study limitations

We acknowledge the following limitations of the study. First, it is a retrospective monocentric study. The workup of findings that were identified ex-post could not be ascertained. Second, most of the insignificant or potentially significant EPFs were not pathologically verified, and the diagnosis was based only on the imaging method. Third, significant EPFs related and unrelated to prostate cancer were analyzed together, because the initial impression on MRI may be incorrect. Last, EPFs were reported by radiologists with various experience in MRI (3–16 years), who were, however, informed of the scope of expected findings before reviewing the scans.

## Conclusions

Extraprostatic findings (EPFs) in MRI of the prostate are frequent, but only 2.4% are clinically significant. One-third of significant EPFs are not reported. Only 30% of potentially significant or significant findings are subject to further workup and 21% influence patient management. Because there is significant variability among radiologists in reporting EPFs, and half of them do not communicate the importance of EPFs, we suggest that an additional “E” category should be attached to the PI-RADS classification similarly to C-RADS, to identify findings that require further workup.

### Supplementary Information

**Additional file 1: Table S1.** Number of findings per Pi-RADS category. **Table S2.** Rare (< 1%) extraprostatic findings that may have clinical significance. **Table S3.** Comparison of reporting extraprostatic findings among four radiologists (R1 – R4). The total number of findings per patient and reporting frequency of common findings. **Table S4.** Workup of significant and potentially significant findings included in the MRI report.

## Abbreviations

C-RADS: CT Colonography Reporting and Data System
EPFs: Extraprostatic findings
MRI: Magnetic resonance imaging
PI-RADS: Prostate Imaging Reporting and Data System
