# Supplementary material for: Quantification and significance of extraprostatic findings on prostate MRI: a retrospective analysis and three-tier classification
Source: Insights Imaging. 2023 Dec 10;14:215. doi: 10.1186/s13244-023-01549-9 (PMC10710974; doi:10.1186/s13244-023-01549-9)
Supplement: Supplementary file 1 — Additional file 1: Table S1. Number of findings per Pi-RADS category. Table S2. Rare (< 1%) extraprostatic findings that may have clinical significance. Table S3. Comparison of reporting extraprostatic findings among four radiologists (R1 – R4). The total number of findings per patient and reporting frequency of common findings. Table S4. Workup of significant and potentially significant findings included in the MRI report. [file 13244_2023_1549_MOESM1_ESM.docx]

**Quantification and significance of extraprostatic findings on prostate MRI: a retrospective analysis and three-tier classification**

**ELECTRONIC SUPPLEMENTARY MATERIAL**

**Suppl. Table 1.** Number of findings per Pi-RADS category

| **PI-RADS category** | **Patients**  n | **Findings**  n | **Findings per patient**  n±SD | **Age**  years±SD |
| --- | --- | --- | --- | --- |
| **PI-RADS ≤2** | 309 | 580 | 1.9±1.1 | 66.8±8.5 |
| **PI-RADS 3** | 64 | 134 | 2.1±1.0 | 67.3±8.4 |
| **PI-RADS 4** | 157 | 301 | 1.9±1.1 | 68.9±7.7 |
| **PI-RADS 5** | 69 | 162 | 2.3±1.3 | 72.0±6.2 |
| **p value** |  |  | 0.0053^1^ | <0.0001^2^ |

^1^Post hoc tests significant for PI-RADS ≤2 vs. PI-RADS 5

^2^Post hoc tests significant for PI-RADS ≤2 vs. PI-RADS 5 and for PI-RADS 3 vs. PI-RADS 5

**Suppl. Table 2.** Rare (<1%) extraprostatic findings that may have clinical significance.

| **Anatomy** | **Condition** | **Frequency** |  | **Non-significant** | **Potentially significant** | **Significant** |  | **Reported** | **Not reported** | **percent** |
| --- | --- | --- | --- | --- | --- | --- | --- | --- | --- | --- |
|  |  | % of 623 pts |  | no. | no. | no. |  | no. | no.^1)^ | % |
| **RARE** | **<1%** |  |  |  |  |  |  |  |  |  |
| MSK | Spondylolisthesis ± spondylolysis | 1.0 |  | 3 | 3 |  |  |  | 6 | 0.0 |
| MSK | Bone marrow diffuse changes | 1.0 |  | 2 | 2 | 2 |  | 3 | 3 (1) | 50.0 |
| G/U | Hydronephrosis, hydroureter | 1.0 |  | 2 | 2 | 2 |  | 3 | 3 (1) | 50.0 |
| MSK | Spinal stenosis secondary | 1.0 |  | 5 | 1 |  |  |  | 6 | 0.0 |
| Bowel | Proctitis | 0.8 |  | 3 | 2 |  |  | 3 | 2 | 60.0 |
| G/U | Seminal vesicle tumor | 0.5 |  |  |  | 3 |  | 3 |  | 100.0 |
| Abd. wall | Parastomic hernia | 0.3 |  | 1 | 1 |  |  | 2 |  | 100.0 |
| Abd. wall | Hernia in scar | 0.3 |  | 1 | 1 |  |  | 1 | 1 | 50.0 |
| Vascular | Dilated pelvis veins | 0.3 |  | 1 | 1 |  |  | 2 |  | 100.0 |
| Bowel | Colon cancer | 0.3 |  |  |  | 2 |  | 2 |  | 100.0 |
| Misc. | Perianal, rectal fistula | 0.3 |  |  | 2 | 1 |  | 2 |  | 100.0 |
| Abd. Wall | Hernia with small bowel | 0.2 |  |  | 1 |  |  | 1 |  | 100.0 |
| Abd. Wall | Hernia with omentum | 0.2 |  |  | 1 |  |  | 1 |  | 100.0 |
| G/U | Bladder coagula | 0.2 |  |  | 1 |  |  | 1 |  | 100.0 |
| Misc. | Schwannoma | 0.2 |  |  | 1 |  |  |  | 1 | 0.0 |
| G/U | Deferent duct inflammation | 0.2 |  |  | 1 |  |  |  | 1 | 0.0 |
| Misc. | Perineal mass | 0.2 |  |  | 1 |  |  | 1 |  | 100.0 |
| G/U | Bladder tumor | 0.2 |  |  |  | 1 |  | 1 |  | 100.0 |
| G/U | Cavernous body thrombosis | 0.2 |  |  |  | 1 |  |  | 1 (1) | 0.0 |

G/U, genitourinary; BM, bone marrow; MSK, musculoskeletal;

^1)^ Number in parentheses denotes unreported significant findings

**Suppl. Table 3.** Comparison of reporting extraprostatic findings among four radiologists (R1 – R4). The total number of findings per patient and reporting frequency of common findings.

| **Reader** | **Protocol** | **Patients** | **Findings per patient** | **Reported findings** | **Diverticulosis** | **Hydrocoele** | **Inguinal hernia (fat)** | **Bladder trabecular hypertrophy** | **Coxartrosis (gr. III and IV)** |
| --- | --- | --- | --- | --- | --- | --- | --- | --- | --- |
| **R1** | P3 | 265 | 1.8±1.0 | 24% | 37 of 123 (30%) | 8 of 95 (8%) | 4 of 23 (17%) | 8 of 29 (28%) | 2 of 25 (8%) |
| **R2** | P3 | 130 | 2.0±1.0 | 50% | 37 of 59 (63%) | 6 of 35 (17%) | 12 of 23 (52%) | 5 of 23 (22%) | 4 of 10 (40%) |
| **R3** | P1, P2 | 110 | 2.5±1.2 | 49% | 25 of 37 (68%) | 25 of 43 (58%) | 4 of 28 (14%) | 12 of 21 (57%) | 8 of 12 (67%) |
| **R4** | P1, P2 | 54 | 2.1±0.9 | 51% | 18 of 27 (67%) | 2 of 14 (14%) | 2 of 10 (20%) | 2 of 8 (25%) | 4 of 4 (100%) |

**Suppl. Table 4.** Workup of significant and potentially significant findings included in the MRI report.

|  | **Yes**  Number (%) | **No**  Number (%) |
| --- | --- | --- |
| **Known before** | 14 (20%) | 57 (80%) |
| **Workup suggested**^1^ | 35 (52%) | 32 (48%) |
| **Workup performed**^1^ | 20 (30%) | 47 (70%) |
| **Changed patient management**^1^ | 14 (21%) | 54 (81%) |

^1^From patients with available follow-up data
